# Out of the Fire, Into the Fog: Association Between Early Mechanical Ventilation and Delirium in Hospitalized Burn Patients

**DOI:** 10.7759/cureus.114981

**Published:** 2026-08-22

**Authors:** Manav M Patel, Michael D Nguyen, Evan S Pistone, Arman Chowdhury, Marc H Phan, Amina El Ayadi, Juquan Song, Michael Erickson

**Affiliations:** 1 Department of Surgery, The University of Texas Medical Branch at Galveston, Galveston, USA

**Keywords:** delirium in icu, icu mortality, mechanical ventilator mortality, trauma burns, trinetx database

## Abstract

Introduction

Delirium is a common and clinically significant complication among hospitalized burn patients, associated with increased morbidity, mortality, and healthcare utilization. While multiple factors contribute to delirium risk, mechanical ventilation has been associated with delirium, although this relationship may reflect underlying illness severity and ICU-level treatment exposure. However, large-scale, multicenter administrative analyses comparing coded outcomes between mechanically ventilated and non-ventilated burn patients remain limited.

Methods

A retrospective cohort study was conducted using the TriNetX (TriNetX, LLC, Cambridge, Massachusetts, United States) database to identify adult hospitalized burn patients with International Classification of Diseases (ICD)-coded burns involving ≥20% total body surface area. Patients were stratified into two cohorts based on receipt of mechanical ventilation within five days of the index burn event. Exclusion criteria included age <18 years and diagnoses of dementia, Alzheimer’s disease, substance use disorders, or other conditions associated with baseline cognitive impairment. Cohorts were propensity score matched for age, sex, race, ethnicity, ICD-coded burn extent history, hypertension, diabetes, ischemic heart disease, sepsis, inhalation injury, and respiratory disorders. Outcomes assessed within 30 days included administratively documented delirium, disorientation, mortality, anxiety disorders, depressive disorders, and acute stress-related disorders.

Results

There were 1,541 patients in each cohort after matching. Ventilated patients had higher rates of administratively documented delirium (risk ratio (RR): 2.16, 95% CI: 1.51-3.09, p<0.0001), mortality (RR: 5.06, 95% CI: 4.23-6.05, p<0.0001), anxiety disorders (RR: 1.88, 95% CI: 1.58-2.21, p<0.0001), disorientation (RR: 3.39, 95% CI: 2.27-5.06, p<0.0001), and acute stress-related disorders (RR: 1.77, 95% CI: 1.40-2.22, p<0.0001). Depressive disorders were not significantly different between cohorts (RR: 1.42, 95% CI: 0.85-2.39, p=0.19).

Discussion

Early mechanical ventilation was associated with higher rates of administratively documented delirium, mortality, anxiety disorders, disorientation, and acute stress-related disorders among adult hospitalized burn patients. However, these findings likely reflect both ICU-level treatment exposure and substantial residual confounding by underlying illness severity. Early mechanical ventilation may identify a high-risk subgroup that warrants structured neurocognitive monitoring, psychological screening, sedation-conscious care, and post-extubation support. Given the observational design and reliance on administrative coding, these findings should be interpreted as hypothesis-generating.

## Introduction

Delirium is a neuropsychiatric condition defined by an acute disturbance in attention, cognition, and awareness attributable to either an underlying medical condition, substance intoxication, withdrawal, or exposure to a toxin. Mechanisms contributing to the pathogenesis of delirium include cerebrovascular dysfunction, neuroinflammation, altered cerebral metabolism, and neurotransmitter imbalances [1]. Associated with increased mortality, morbidity, prolonged ICU stay, and long-term cognitive impairment, delirium remains challenging to treat and has poor outcomes, especially among burn patients [2-4].

There is a higher incidence of delirium and predicted probability to develop delirium in burn patients when compared to other surgical demographics. In 2024, a meta-analysis reported a 20.5% incidence of delirium in burn patients [5]. Part of the higher incidence of delirium is likely due to the uniqueness of burn pathophysiology, which is well known to be associated with hypermetabolic states [6-8]. A hypermetabolic state in conjunction with compromised thermoregulation, fluid regulation, and electrolyte regulation is a unique disease state that creates a high risk for the development of delirium in burn patients [6-8]. Currently, burns involving ≥20% total body surface area are generally accepted as severe burns and are known to cause a hypermetabolic state [8]. Another challenge is the need for long-term sedation, frequent surgeries, and mechanical ventilation in critically ill patients [9,10]. Prolonged analgesia and sedation may also mask or mimic the effects of delirium, making proper diagnosis particularly difficult for ICU teams [9,10]. Deep sedation and mechanical ventilation may also create barriers to delirium screening, as patients receiving life-support often have altered arousal, limited ability to participate in bedside assessments, and greater underlying illness severity [9]. Given the multifactorial and often unmodifiable nature of delirium risk in burn patients, early recognition through reliable diagnostic tools becomes essential in guiding appropriate management.

Currently, the early identification and management of risk factors remain the best approach to diagnosing, preventing, and treating delirium [11]. In the ICU, the Confusion Assessment Method for the ICU (CAM-ICU) is commonly used and is the most reliable, validated scale to date [12,13]. Delirium may present in either hyperactive, hypoactive, or mixed forms, which requires the use of models such as the CAM-ICU to properly diagnose. However, the CAM-ICU relies on the provider to know whether the patient has an acute change from mental status baseline to whom baseline may not be known. Furthermore, it requires that patients not be heavily sedated to properly score, which may not be possible for in-patient populations that typically require extensive sedation like burn patients. As a result, the identification of risk factors is ever more important in delirium management.

Risk factors for delirium can be broadly categorized into modifiable and non-modifiable. Non-modifiable risk factors include advanced age, dementia, prior coma, pre-ICU emergency surgery or trauma, higher American Society of Anesthesiologists (ASA) scores, total body surface area (TBSA) burned >10%, ICU admission, and higher ICU length of stay (LOS) [10,14,15]. Common modifiable risk factors include benzodiazepine use, pain, blood transfusions, increased injury to treatment time, surgical frequency, opioid infusions, infections, and use of restraints [10,14,15].

Recent evidence has identified an association between mechanical ventilation and delirium in burn patients, with patients requiring mechanical ventilation reported to have higher odds of delirium than those who do not [16]. However, mechanical ventilation may also represent a marker of overall illness severity rather than a direct causal factor, reflecting underlying physiologic instability or need for intensive care. Existing studies evaluating this relationship are often limited by single-center designs or smaller cohort sizes. Large multicenter analyses examining this association in burn patients remain limited. To address this gap, we conducted a retrospective cohort study using the TriNetX (TriNetX, LLC, Cambridge, Massachusetts, United States) database to evaluate adult patients with severe burns involving ≥20% total body surface area. We compared patients who underwent mechanical ventilation within five days of injury to those who did not, assessing 30-day outcomes including administratively documented delirium, disorientation, anxiety disorders, depressive disorders, acute stress-related disorders, and mortality.

The primary objective of this study was to evaluate the association between early mechanical ventilation within five days of burn injury and 30-day administratively documented delirium among adult hospitalized burn patients in the TriNetX database. Secondary objectives included assessment of 30-day mortality, disorientation, anxiety disorders, depressive disorders, and acute stress-related disorders. Because TriNetX does not provide granular bedside data such as CAM-ICU results, sedation depth, ventilator settings, or detailed physiologic severity markers, this study was designed as an administrative database analysis of coded outcomes rather than a true clinical incidence study of delirium.

## Materials and methods

We conducted a retrospective cohort study using the TriNetX database to identify hospitalized burn patients from 2005 to 2025 who received either mechanical ventilation or non-ventilated care within five days of the index burn event. The TriNetX Research Network (https://trinetx.com) is a database that provides de-identified medical records of approximately 113 million patients from 64 healthcare organizations (HCOs), including diagnoses (International Classification of Diseases, Tenth Revision, Clinical Modification (ICD-10-CM) codes), procedures (ICD-10-PCS (International Classification of Diseases, Tenth Revision, Procedure Coding System) or Current Procedural Terminology (CPT) codes), medications (RxNorm codes), and laboratory values (Logical Observation Identifiers Names and Codes (LOINC)). Data are aggregated across participating institutions and reflect routine clinical documentation and coding practices. TriNetX only consists of de-identified patient information from proprietary participating HCOs to ensure anonymity. Users do not have access to any protected health information or personal data. For this study, institutional review board approval was not required as no protected health information or data is made available. Individual data within cohorts cannot be tracked across years, and the data is not able to be traced to a specific partner on the network.

**Figure 1:**
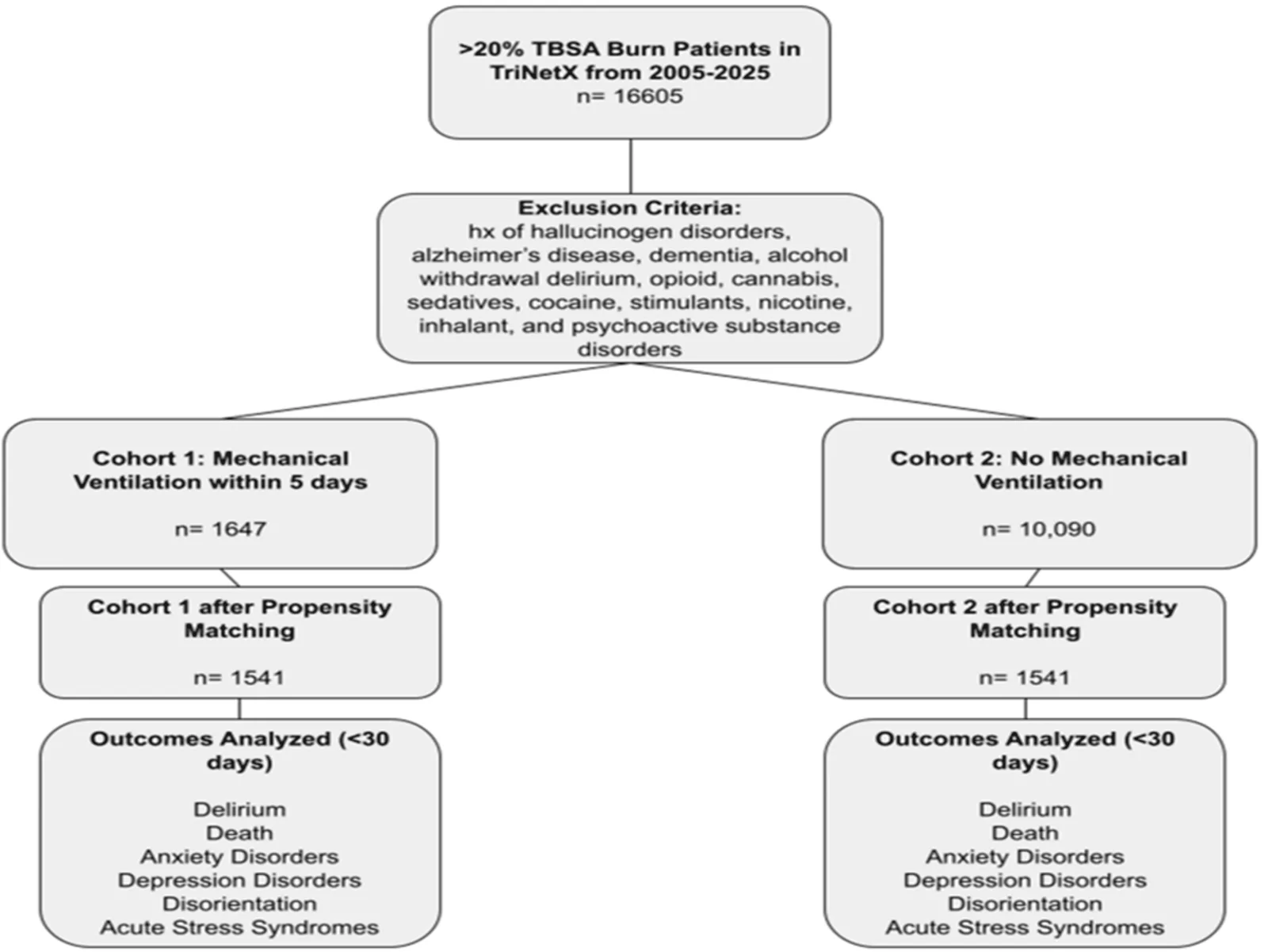
Cohort Construction Flowchart TBSA: total body surface area, hx: history.

Key inclusion criteria were burn patients aged 18-99 with an index burn injury involving ≥20% TBSA, identified using ICD-10 codes T31.2-T31.9, from 2005 to 2025 (Table 1). Additional ICD-coded burn extent categories, including burns involving <20% TBSA, were used only as covariates to characterize prior or concurrent burn extent coding history during propensity score matching and were not used as inclusion criteria. Exclusion criteria included age under 18 years and diagnoses of dementia, Alzheimer’s disease, hallucinogen-related disorders, alcohol-induced delirium, or substance use disorders. Patients were divided into two cohorts based on whether they received mechanical ventilation within five days of the burn event. Mechanical ventilation was defined using ICD-10-PCS procedure codes recorded within five days of the index burn encounter and was analyzed as a binary exposure because the primary objective of this study was to evaluate whether receipt of early mechanical ventilation identified a higher-risk subgroup among hospitalized burn patients, rather than to evaluate a dose-response relationship by duration of ventilation. The ICD-10-PCS codes used to define mechanical ventilation include respiratory ventilation lasting <24 consecutive hours, 24-95 consecutive hours, and >96 consecutive hours. Therefore, this exposure definition includes heterogeneous clinical scenarios, ranging from brief perioperative ventilation to prolonged critical care ventilation. This heterogeneity was considered a limitation of the exposure definition. The five-day window was selected to capture early respiratory failure related to the initial burn injury and acute resuscitation period, while minimizing inclusion of delayed complications or unrelated respiratory events. Thus, Cohort 1 included mechanically ventilated patients and Cohort 2 included non-ventilated patients (Figure 1).

**Table 1:** Codes for Cohort Construction *These three ICD-10-PCS ventilation-duration categories were combined into a single early mechanical ventilation exposure group for the primary analysis. ICD-10-PCS: International Classification of Diseases, Tenth Revision, Procedure Coding System.

Inclusion	
Burns involving ≥20% of body surface	T31.2-T31.9
Mechanical Ventilation Exposure Codes Included in Binary Ventilated Cohort*	
Respiratory ventilation (<24 consecutive hours)	5A1935Z
Respiratory ventilation (24-95 consecutive hours)	5A1945Z
Respiratory ventilation (>96 consecutive hours)	5A1955Z
Exclusion	
Alzheimer’s disease	G30
Dementia	F02
Alcohol dependence w/ withdrawal delirium	F10.231
Opioid disorders	F11
Cannabis disorders	F12
Sedative, hypnotic, or anxiolytic disorders	F13
Cocaine disorders	F14
Stimulant disorders	F15
Hallucinogen use disorders	F16
Nicotine dependence	F17
Inhalant use disorders	F18
Psychoactive substance disorders	F19
Propensity Score Matching Covariates	
Burns involving ≥20% of body surface	T31.2-T31.9
Essential (primary) hypertension	I10
Type 2 diabetes mellitus	E11
Chronic ischemic heart disease	I25
Sepsis, specified organism	A41.89
Sepsis, unspecified organism	A41.9
Smoke inhalation Injury	J70.5
Respiratory disorders	J98

All outcomes were identified using ICD-10 diagnostic codes (Table 2). Delirium was defined using ICD-10 diagnostic coding and was not confirmed using structured bedside screening tools such as the Confusion Assessment Method for the ICU (CAM-ICU). Therefore, this outcome reflects administratively documented delirium rather than true bedside delirium incidence. Given the known reliance of delirium diagnosis on active clinical screening, ICD-10 coding likely undercaptures the true frequency of delirium in this population. Disorientation and acute stress-related disorders were similarly identified using administrative diagnostic codes, which may overlap clinically with delirium or represent related neuropsychiatric conditions. Disorientation was included as a proxy neurocognitive symptom given the limitations of coding-based delirium detection. As such, these outcomes should be interpreted within the limitations of coding-based definitions rather than standardized bedside assessment tools.

**Table 2:** Codes for Outcome Definitions

Outcome	Description	ICD-10 Code(s)
Administratively Documented Delirium	Acute disturbance in attention, awareness, and cognition	F05
Disorientation	Altered mental status or confusion not otherwise specified	R41.0
Anxiety Disorders	Anxiety-related disorders including generalized anxiety and related conditions	F41, F40-F48
Depressive Disorders	Depressive disorders including major depressive disorder and related conditions	F32.A
Acute Stress-Related Disorders	Acute stress reaction following traumatic injury	F43, F43.0, F43.10, F43.11
Mortality	All-cause mortality within 30 days of index event	TriNetX mortality indicator

All analysis was conducted within the TriNetX platform. Propensity score estimation and matching were performed within the TriNetX platform using its built-in matching algorithm. A 1:1 greedy nearest-neighbor algorithm with a caliper of 0.1 pooled standard deviation was used to reduce measured baseline differences between cohorts. The platform does not provide all implementation-level modeling details; therefore, we did not specify logistic regression unless directly provided by the platform output. Cohorts were propensity score matched for age, sex, race, ethnicity, ICD-coded burn extent history, hypertension, diabetes, ischemic heart disease, sepsis, inhalation injury, and respiratory disorders. Balance was assessed using standardized mean differences (SMDs), with values <0.1 considered acceptable. However, some matched variables, including sepsis, inhalation injury, and respiratory disorders, may reflect illness severity or lie along the causal pathway between burn injury, mechanical ventilation, and outcomes. Therefore, matching should be interpreted as reducing measured baseline differences rather than establishing causality.

Sedation exposure could not be reliably captured using available procedure-code data in this analysis. An initial attempt to identify deep sedation/general anesthesia using procedural codes produced clinically implausible frequencies and was therefore not included in the final matching model or baseline table. Medication-level sedative exposure, sedation depth, and sedation duration were not available with sufficient granularity for this analysis. This is an important limitation because sedative and analgesic medications are established contributors to delirium risk in mechanically ventilated ICU patients. Despite propensity score matching, residual confounding by indication remains likely, as mechanical ventilation is frequently initiated in the setting of greater illness severity, including respiratory failure, airway compromise, or systemic instability. Key markers of severity such as Glasgow Coma Scale, vasopressor requirement, ventilator duration, and severity of inhalation injury were not available within the dataset and therefore could not be accounted for in the matching process.

Patients were evaluated within 30 days for administratively documented delirium, mortality, anxiety disorders, depressive disorders, disorientation, and acute stress-related disorders using risk ratios (RRs) with corresponding 95% confidence intervals (CIs). Outcomes were identified using ICD-10 diagnostic codes recorded within the TriNetX network (Table 2). Mortality was defined as all-cause mortality within 30 days of the index event as captured within the TriNetX platform. Patients with documented outcomes prior to the index window were excluded from outcome-specific analyses where applicable. These comparisons between groups were performed using the TriNetX built-in risk analysis module, which uses large sample normal approximation (z) tests to estimate differences in proportions. Statistical significance was defined as p<0.05.

## Results

Patient demographics, comorbidities, and ICD-coded burn extent history

Before propensity matching, 1,647 patients were in Cohort 1 (mechanically ventilated) and 10,087 patients in Cohort 2 (non-ventilated). There were significant differences in age, sex, race, and comorbidities. Patients in Cohort 1 were older on average (mean age: 62.7 years vs 58.4 years, p<0.001) and male (71.2% vs 66.3%, p<0.001). Racial demographics also differed, with White patients being more ventilated on average (58.9% vs 56.6%, p<0.05). Comorbidities such as hypertension (9.1% vs 9.1%, p=0.97), type 2 diabetes (4.3% vs 4.4%, p=0.77), and chronic ischemic heart disease (2.6% vs 2.4%, p=0.66) were similar between groups before matching, but there were differences in respiratory disorders (6.3% vs 4.4%, p<0.001) and sepsis (4.2% vs 1.2%, p<0.001). ICD-coded burn extent history also differed before matching. Patients in the ventilated cohort were more likely to have codes for burns involving 20%-49% TBSA, while patients in the non-ventilated cohort were more likely to have additional codes for burns involving <20% TBSA. These <20% TBSA categories were used as matching covariates reflecting burn extent coding history and did not define study inclusion.

These pre-matching comparisons are descriptive and should not be used for outcome interpretation because the groups differed substantially before matching. After propensity matching, 1,541 patients remained in each cohort. Matching improved balance across measured covariates, including age, sex, race, ethnicity, and major comorbidities. Residual differences in selected variables may persist and are reflected in Table 3. Standardized mean differences (SMDs) were used to assess balance. Most variables demonstrated improved comparability after matching, although residual imbalance persisted for selected variables, including ICD-coded lower TBSA categories and hypertension, as reflected in Table 3.

**Table 3:** Baseline Characteristics Before and After Propensity Score Matching Continuous variables were compared using Student’s t-test and categorical variables using chi-square tests. Standardized mean differences (SMDs) are reported to assess covariate balance after propensity score matching, with values <0.1 indicating adequate balance. Test statistic values are not displayed as they are not provided by the TriNetX platform. Note: Study inclusion was based on ICD-10 codes T31.2-T31.9 for burns involving ≥20% TBSA. Burn extent categories involving <20% TBSA were included only as covariates to characterize ICD-coded burn extent history during propensity score matching and were not used as inclusion criteria. TBSA: total body surface area, ICD = International Classification of Diseases.

Characteristic	Cohort 1 Before: n (%)	Cohort 2 Before: n (%)	Before: p-Value	Standardized Mean Difference	Cohort 1 After: n (%)	Cohort 2 After: n (%)	After: p-Value	Standardized Mean Difference
Demographics								
Male	1,172 (71.2%)	6,692 (66.3%)	<0.001	0.10	1,096 (71.1%)	1,093 (70.9%)	0.91	0.01
Female	443 (26.9%)	3,230 (32.0%)	<0.001	0.20	417 (27.1%)	425 (27.6%)	0.75	0.02
White	970 (58.9%)	5,718 (56.7%)	0.09	0.04	910 (59.1%)	895 (58.1%)	0.58	0.02
Black or African American	240 (14.6%)	1,620 (16.1%)	0.13	0.11	220 (14.3%)	206 (13.4%)	0.46	0.01
Hispanic or Latino	135 (8.2%)	1,188 (11.8%)	<0.001	0.04	124 (8.1%)	126 (8.2%)	0.90	0.03
Not Hispanic or Latino	1,106 (67.2%)	5,819 (57.7%)	<0.001	0.12	1,024 (66.5%)	1,006 (65.3%)	0.49	0.01
Asian	52 (3.2%)	294 (2.9%)	0.59	0.01	51 (3.3%)	45 (2.9%)	0.53	0.02
American Indian or Alaska Native	31 (1.9%)	71 (0.7%)	<0.001	0.10	25 (1.6%)	31 (2.0%)	0.42	0.03
Native Hawaiian or Other Pacific Islander	15 (0.9%)	79 (0.8%)	0.59	0.01	15 (1.0%)	21 (1.4%)	0.31	0.04
Comorbidities								
Essential (primary) hypertension	150 (9.1%)	922 (9.1%)	0.97	0.01	125 (8.1%)	80 (5.2%)	<0.001	0.12
Type 2 diabetes mellitus	70 (4.3%)	445 (4.4%)	0.77	0.01	53 (3.4%)	37 (2.4%)	0.09	0.06
Chronic ischemic heart disease	43 (2.6%)	245 (2.4%)	0.66	0.01	35 (2.3%)	22 (1.4%)	0.08	0.06
Other respiratory disorders	104 (6.3%)	443 (4.4%)	<0.001	0.09	80 (5.2%)	61 (4.0%)	0.10	0.06
Complications								
Sepsis, unspecified organism	69 (4.2%)	122 (1.2%)	<0.001	0.18	39 (2.5%)	27 (1.8%)	0.14	0.05
Other specified sepsis	10 (0.6%)	10 (0.1%)	<0.001	0.09	10 (0.7%)	10 (0.7%)	1.00	0.01
Respiratory conditions due to smoke inhalation	29 (1.8%)	20 (0.2%)	<0.001	0.16	13 (0.8%)	11 (0.7%)	0.68	0.02
ICD-coded burn extent history								
Burns involving less than 10% of body surface	26 (1.6%)	567 (5.6%)	<0.001	0.22	20 (1.3%)	106 (6.9%)	<0.001	0.28
Burns involving 10%-19% of body surface	27 (1.6%)	520 (5.2%)	<0.001	0.20	21 (1.4%)	81 (5.3%)	<0.001	0.22
Burns involving 20%-29% of body surface	101 (6.1%)	47 (0.5%)	<0.001	0.32	52 (3.4%)	44 (2.9%)	0.41	0.03
Burns involving 30%-39% of body surface	83 (5.0%)	45 (0.5%)	<0.001	0.28	47 (3.1%)	37 (2.4%)	0.27	0.04
Burns involving 40%-49% of body surface	60 (3.6%)	28 (0.3%)	<0.001	0.24	34 (2.2%)	26 (1.7%)	0.30	0.04
Burns involving 50%-59% of body surface	35 (2.1%)	25 (0.3%)	<0.001	0.17	22 (1.4%)	16 (1.0%)	0.33	0.04
Burns involving 60%-69% of body surface	23 (1.4%)	25 (0.3%)	<0.001	0.13	17 (1.1%)	14 (0.9%)	0.59	0.02
Burns involving 70%-79% of body surface	13 (0.8%)	16 (0.2%)	<0.001	0.09	10 (0.7%)	10 (0.7%)	1.00	0.01
Burns involving 80%-89% of body surface	14 (0.9%)	21 (0.2%)	<0.001	0.09	11 (0.7%)	12 (0.8%)	0.83	0.01
Burns involving 90% or more of body surface	12 (0.7%)	10 (0.1%)	<0.001	0.10	10 (0.7%)	10 (0.7%)	1.00	0.01

Outcomes

Outcomes prior to the time window were excluded. A summary of 30-day outcomes following propensity score matching is presented in Table 4. Within 30 days, an ICD-coded diagnosis of delirium was recorded in 5.9% of ventilated patients compared to 2.7% of non-ventilated patients (absolute risk difference: 3.16%, RR: 2.16, 95% CI: 1.51-3.09, p<0.001). Mortality was substantially higher in the ventilated cohort, occurring in 41.1% of patients versus 8.1% in the non-ventilated cohort (absolute risk difference: 32.98%). This corresponded to a risk ratio of 5.06 (95% CI: 4.23-6.05, p<0.001). Anxiety disorders were identified in 22.6% of ventilated patients compared to 12.1% of non-ventilated patients (RR: 1.88, 95% CI: 1.58-2.21, p<0.001). Depressive disorders occurred in 2.2% of ventilated patients and 1.6% of non-ventilated patients. The difference was not statistically significant (RR: 1.42, 95% CI: 0.85-2.39, p=0.19). Disorientation occurred in 6.6% of ventilated patients compared to 2.0% of non-ventilated patients (RR: 3.39, 95% CI: 2.27-5.06, p<0.001). Acute stress-related disorders were also significantly more common in the ventilated cohort compared to the non-ventilated cohort, with a RR of 1.77 (95% CI: 1.40-2.22, p<0.001). Absolute risk differences were 3.16% for administratively documented delirium, 4.66% for disorientation, 10.54% for anxiety disorders, 0.66% for depressive disorders, 5.29% for acute stress-related disorders, and 32.98% for mortality. The approximate number needed to harm was 32 for administratively documented delirium, 22 for disorientation, 10 for anxiety disorders, 19 for acute stress-related disorders, and 3 for mortality. Number needed to harm was not reported for depressive disorders because the between-group difference was not statistically significant.

**Table 4:** Thirty-Day Clinical Outcomes After Propensity Score Matching Outcomes were assessed within 30 days of the index event. Absolute risk difference, representing absolute risk increase in this table, was calculated as the event rate in the mechanically ventilated cohort minus the event rate in the non-ventilated cohort. NNH was calculated as 1 divided by the absolute risk difference and rounded up to the nearest whole number. NNH was not reported for depressive disorders because the between-group difference was not statistically significant. NNH: number needed to harm.

Outcome	Ventilated n (%)	Non-Ventilated n (%)	Absolute Risk Difference	Approximate NNH	Risk Ratio (95% CI)	p-Value
Administratively Documented Delirium	90 (5.89%)	42 (2.73%)	3.16%	32	2.16 (1.51-3.09)	<0.001
Disorientation	101 (6.61%)	30 (1.95%)	4.66%	22	3.39 (2.27-5.06)	<0.001
Anxiety Disorders	330 (22.63%)	175 (12.09%)	10.54%	10	1.88 (1.58-2.21)	<0.001
Depressive Disorders	34 (2.23%)	24 (1.57%)	0.66%	Not reported	1.42 (0.85-2.39)	0.19
Acute Stress-Related Disorders	184 (12.21%)	103 (6.92%)	5.29%	19	1.77 (1.40-2.22)	<0.001
Mortality	625 (41.11%)	123 (8.13%)	32.98%	3	5.06 (4.23-6.05)	<0.001

## Discussion

Mechanical ventilation has been associated with delirium in multiple studies of mechanically ventilated adults [13,14]. Prior studies have reported mechanical ventilation as a risk factor for delirium even after adjustment for measured covariates, although residual confounding by illness severity remains difficult to eliminate [13-17]. The relationship between mechanical ventilation and delirium is likely multifactorial. Sedative polypharmacy, particularly the use of multiple intravenous sedative agents, may mediate part of this risk [17]. Experimental and clinical literature has also suggested biologically plausible pathways through which mechanical ventilation and associated ICU exposures may contribute to neurologic dysfunction, including neuroinflammation, neuronal apoptosis, and disruption of the blood-brain barrier, with particularly notable effects in the hippocampus [18,19]. These mechanisms have been hypothesized to occur through aberrant vagus signaling and D2 receptor activation [18]. Other clinical factors that may amplify the risk of delirium in mechanically ventilated patients include advanced age, hypertension, sepsis, hypoalbuminemia, and the use of physical restraints [13,15,16].

The pathophysiologic stress of burn injury may further increase vulnerability to delirium and neurocognitive dysfunction. Severe burns initiate a persistent hypermetabolic state characterized by high energy expenditure, hyperglycemia, and profound systemic inflammation [6-8]. In burn patients, blood-brain barrier disruption and a primed neuroinflammatory state may make the central nervous system more susceptible to secondary insults [20-22]. Mechanical ventilation and associated ICU exposures may plausibly interact with burn-related systemic inflammation, oxidative and nitrosative stress, cerebral edema, and neuronal injury, all of which have been implicated in acute delirium and long-term cognitive impairment [19-24]. Thus, the intersection of burn-induced hypermetabolism, systemic inflammation, critical illness, sedation exposure, and mechanical ventilation may create an environment that heightens vulnerability to neurocognitive dysfunction. In this context, mechanical ventilation may represent an additional physiologic stressor or, alternatively, a marker of more severe systemic illness.

In two propensity-matched burn cohorts separated by mechanical ventilation use, we identified higher rates of administratively documented delirium in mechanically ventilated patients compared with burn patients who did not receive mechanical ventilation. This is consistent with prior research identifying mechanical ventilation as a risk factor for delirium in severe burn patients [16]. With high rates of psychological sequelae found in burn patients, this finding is not unusual [25-27]. However, rates in the literature are substantially higher than the 5.9% reported in our TriNetX analysis, with some studies reporting delirium rates in mechanically ventilated burn patients as high as 80% [10]. In contrast, the absolute rate of administratively documented delirium in our study was notably lower, suggesting substantial under-detection. This discrepancy is most likely attributable to reliance on ICD-10 diagnostic coding rather than structured bedside screening tools such as CAM-ICU. Delirium is frequently underdiagnosed and undercoded in clinical practice, particularly in critically ill, sedated, or mechanically ventilated patients, where accurate assessment is challenging. Therefore, the absolute rate of delirium reported in this study should not be interpreted as the true clinical incidence of delirium among mechanically ventilated burn patients. Rather, it reflects the frequency of administratively documented delirium within the TriNetX network. This likely biases absolute incidence estimates downward and may affect the reliability of effect estimates. The observed relative differences should therefore be interpreted cautiously and considered hypothesis-generating.

As a retrospective study of electronic health records, the value of our data comes from the ability to evaluate a large multicenter population and identify broad associations that may warrant further prospective study. However, coding and diagnostic limitations are central to the interpretation of these findings. Clinicians may recognize delirium at the bedside but fail to enter a formal diagnosis into the medical record. Even when delirium is noted clinically, it may not be coded, especially if it is transient, attributed to sedation or critical illness, or overshadowed by other acute problems [12,28]. Accurate delirium diagnosis requires structured screening, typically with tools such as CAM-ICU [3,12,28]. However, CAM-ICU use may be inconsistent across centers. Accurate delirium diagnosis may also be challenging in burn ICUs due to frequent deep sedation, intubation, paralysis, and lack of a known baseline mental status, which can result in under-recognition and delayed intervention [10,12,28]. Therefore, the higher rate of administratively documented delirium among ventilated patients remains a clinically significant finding that warrants cautious interpretation and further study.

In addition to administratively documented delirium, other coded neuropsychiatric outcomes selected for analysis included disorientation, acute stress-related disorders, anxiety disorders, and depression. Mechanically ventilated patients had higher rates of coded disorientation, anxiety disorders, and acute stress-related disorders compared with non-ventilated patients. However, these findings should be interpreted cautiously because coded neuropsychiatric outcomes are susceptible to detection and documentation bias in electronic health record (EHR)-based studies [16,25-27]. Patients requiring mechanical ventilation receive more intensive monitoring, repeated clinical assessments, and more extensive documentation, which may increase the likelihood that neuropsychiatric symptoms are recognized and coded. Likewise, mechanically ventilated patients in the ICU, regardless of underlying diagnosis, may experience psychological distress, including anxiety, depression, and post-ICU psychiatric morbidity, as highlighted by large cohort studies [29]. Depression was numerically higher in the ventilated cohort but was not statistically significant and should not be interpreted as significantly associated with mechanical ventilation in this study. Disorientation should not be interpreted as equivalent to delirium, as it may reflect medication effects, hypoxia, metabolic encephalopathy, sedation, baseline cognitive impairment, or other causes of altered mental status. Overall, these findings suggest that mechanically ventilated burn patients represent a population at increased risk for coded neuropsychiatric complications, although the observed associations may reflect both true clinical burden and differences in surveillance, documentation, and underlying illness severity. Improved observation and psychological screening may help identify these complications earlier and guide appropriate intervention.

Mortality was also evaluated as a secondary outcome because it is more reliably captured in electronic health records than transient neuropsychiatric diagnoses. The markedly higher 30-day mortality observed among mechanically ventilated patients should not be interpreted as evidence that mechanical ventilation independently causes mortality. Rather, early mechanical ventilation likely identifies a subgroup of burn patients with profound physiologic instability. Mechanical ventilation is often required in the setting of airway compromise, severe inhalation injury, acute respiratory failure, acute respiratory distress syndrome, shock, or multiorgan dysfunction. Although propensity score matching adjusted for available comorbidities and ICD-coded burn extent, the database did not capture several key determinants of mortality, including burn depth, inhalation injury severity, vasopressor requirement, ventilator settings, duration of ventilation, Glasgow Coma Scale, lactate, organ failure severity, or ICU length of stay. These unmeasured variables likely explain a substantial portion of the observed mortality association. Therefore, residual confounding by indication is central to the interpretation of mortality in this study.

As a large multicenter database study, this analysis has several limitations. First, there may be underreporting and misclassification bias due to the use of administrative coding to define both exposures and outcomes. Mechanical ventilation was treated as a binary exposure despite ICD-10-PCS codes distinguishing <24 hours, 24-95 hours, and >96 hours of respiratory ventilation. As a result, patients receiving brief perioperative intubation may have been grouped with patients requiring prolonged ventilation for respiratory failure, acute respiratory distress syndrome, inhalation injury, or multiorgan dysfunction. This exposure heterogeneity may introduce misclassification and limits the ability to determine whether observed associations differ by ventilation duration. Sedation exposure was also not reliably captured. This is a major limitation because sedative and analgesic medications, including benzodiazepines, propofol, opioids, and other ICU medications, may independently influence delirium risk and may differ substantially between ventilated and non-ventilated patients. Similarly, delirium and other neuropsychiatric outcomes depend on clinician recognition and documentation, leading to likely underreporting. Therefore, the observed delirium risk ratio should be interpreted as an association with coded delirium rather than a reliable estimate of the true clinical delirium risk ratio. Second, residual confounding by indication remains central to the interpretation of this study. Despite propensity score matching, important clinical variables such as inhalation injury severity, Glasgow Coma Scale, vasopressor requirement, nutritional status, depth of sedation, ventilator duration, ventilator settings, lactate, organ failure severity, and ICU length of stay were not available and therefore could not be accounted for. As a result, mechanical ventilation may act as a proxy for underlying disease severity rather than an independent causal factor, particularly for mortality. Additionally, ICD-coded TBSA and burn extent history do not fully capture physiologic instability, burn depth, burn etiology, inhalation injury severity, procedural burden, or the need for intubation, further limiting risk stratification. Reliance on TriNetX-participating institutions and ICD-coded severe burn criteria may also introduce selection bias by preferentially capturing patients with more complete documentation or coding of burn extent and critical illness. Lastly, as with all federated database studies, aggregation of data across multiple institutions with heterogeneous clinical practices may introduce variability and limit generalizability.

Despite these limitations, the findings from this study may help identify burn patients who warrant closer neurocognitive and psychological monitoring. Because this analysis did not stratify outcomes by ventilation duration, the findings should not be interpreted as showing equivalent risk across brief perioperative ventilation and prolonged critical care ventilation. Given the association between early mechanical ventilation and administratively documented delirium in burn patients, clinicians should maintain a high index of suspicion and use validated tools like CAM-ICU as consistently as possible, even in sedated or intubated patients. Neurocognitive monitoring is imperative and modified screening protocols may be necessary in the burn ICU setting to overcome known barriers to assessment. Receipt of early mechanical ventilation may serve as a practical marker to prompt neurocognitive risk assessment, including plans for delirium monitoring, psychological screening, and post-extubation support, given the higher rates of administratively documented delirium, anxiety disorders, disorientation, and acute stress-related disorders observed in this study. Additionally, burn units should seek to optimize analgesia and sedation regimens and minimize sedative polypharmacy when clinically feasible. While mechanical ventilation is often necessary and lifesaving, its use may identify patients who are at elevated risk for neuropsychiatric complications due to the combined effects of burn severity, critical illness, sedation exposure, and ICU-level care. Future prospective studies using validated delirium screening tools, granular sedation data, ventilation duration, burn depth, inhalation injury severity, and ICU severity markers are needed to clarify the relationship between mechanical ventilation and neuropsychiatric outcomes in burn patients.

## Conclusions

Delirium is an important complication among hospitalized burn patients and is associated with morbidity, prolonged recovery, and increased healthcare utilization. In this retrospective TriNetX study, early mechanical ventilation was associated with higher 30-day rates of administratively documented delirium, mortality, anxiety disorders, disorientation, and acute stress-related disorders among adult hospitalized burn patients, while depressive disorders were not significantly different between cohorts. These findings should be interpreted as hypothesis-generating associations within an administrative database rather than evidence of causality or true clinical delirium incidence. Mechanical ventilation likely identifies a high-risk subgroup with greater underlying illness severity, and residual confounding by indication remains central to interpretation. Future prospective studies incorporating validated delirium screening, sedation exposure, ventilation duration, burn depth, inhalation injury severity, and ICU severity markers are needed to clarify the relationship between mechanical ventilation and neuropsychiatric outcomes in burn patients.
